# Sulfated Galactofucan from the Brown Alga *Saccharina latissima*—Variability of Yield, Structural Composition and Bioactivity

**DOI:** 10.3390/md13010076

**Published:** 2014-12-26

**Authors:** Karina Ehrig, Susanne Alban

**Affiliations:** Pharmaceutical Institute, Christian-Albrechts-University of Kiel, Gutenbergstrasse 76, 24118 Kiel, Germany; E-Mail: kehrig@pharmazie.uni-kiel.de

**Keywords:** *Saccharina latissima*, brown alga, fucoidan, sulfated polysaccharides, galactofucan, habitat, harvest time, salinity, tidal amplitude

## Abstract

The fucose-containing sulfated polysaccharides (SP) from brown algae exhibit a wide range of bioactivities and are, therefore, considered promising candidates for health-supporting and medicinal applications. A critical issue is their availability in high, reproducible quality. The aim of the present study was to fractionate and characterize the SP extracted from *Saccharina latissima* (S.l.-SP) harvested from two marine habitats, the Baltic Sea and North Atlantic Ocean, in May, June and September. The fractionation of crude S.l.-SP by anion exchange chromatography including analytical investigations revealed that S.l.-SP is composed of a homogeneous fraction of sulfated galactofucan (SGF) and a mixture of low-sulfated, uronic acid and protein containing heteropolysaccharides. Furthermore, the results indicated that S.l. growing at an intertidal zone with high salinity harvested at the end of the growing period delivered the highest yield of S.l.-SP with SGF as the main fraction (67%). Its SGF had the highest degree of sulfation (0.81), fucose content (86.1%) and fucose/galactose ratio (7.8) and was most active (e.g., elastase inhibition: IC_50_ 0.21 μg/mL). Thus, S.l. from the North Atlantic harvested in autumn proved to be more appropriate for the isolation of S.l.-SP than S.l. from the Baltic Sea and S.l. harvested in spring, respectively. In conclusion, this study demonstrated that habitat and harvest time of brown algae should be considered as factors influencing the yield as well as the composition and thus also the bioactivity of their SP.

## 1. Introduction

Sulfated polysaccharides (SP) represent a complex group of biopolymers with a wide range of important biological functions and activities [1,2,3,4,5]. Besides the sulfated glycosaminoglycans of vertebrates, SP are ubiquitous components of marine algae and marine invertebrates. While carrageenans and agarans, two types of sulfated galactans extracted from red algae species, have been industrially applied as hydrocolloids for many decades [6], fucoidans, the typical SP of brown algae of the class Phaeophyceae, are increasingly attracting attention as promising candidates for numerous health-supporting and therapeutic applications [3,7,8,9]. The recent interest has mainly focused on their potentially beneficial effects in humans including antitumor, immunomodulatory, anti-inflammatory, antiviral, antithrombotic, anticoagulant, and antioxidant effects, as well as specific activities against kidney, liver and urinary system disorders [7,10]. However, despite intense research during the last two decades, there is thus far no approved medicinal product containing fucoidan or any other marine SP as drug substance; they are currently only utilized as ingredients in food supplements and cosmetics [8,11,12].

Critical issues for marine SP are the high requirements on the pharmaceutical quality of medicinal products, which is essential for their efficacy and safety [11]. It is generally challenging to produce marine SP in a reproducible quality, since they are not only usually complex, heterogeneous molecule mixtures, but they also vary substantially in their composition depending on the source material (e.g., alga species, harvest time), environmental parameters (e.g., light, nutrition, salinity, temperature), as well as the process of extraction and purification [10,11,13,14]. Particularly, the fucoidans found in the cell walls and intercellular spaces of brown algae represent a tremendous number of structurally distinct fucose-containing SP ranging from homofucans to complex, highly branched heteropolysaccharides [3,10,15,16] so that some authors consider the term fucose-containing sulfated polysaccharides more appropriate than the term fucoidan [10,17]. Even crude fucoidan isolated from a single species of brown algae mostly consists of a mixture of structurally distinct polymers and the composition of this mixture may considerably vary depending on a multitude of factors (see above).

Since the structure of SP has an important impact on its biological activities, it is, therefore, impossible to generalize any reported activities of a tested fucoidan, especially if its structural composition is not indicated. Aggravating this situation, the compounds indicated in literature as “fucoidans” considerably vary in their degree of purity, *i.e*., their content of co-extracted compounds like laminarin, alginic acid, proteins, polyphenols, *etc*. may influence the observed biological effects.

The present study was part of the national cooperation project “Algae against Cancer” with the aim to identify compounds with antitumor activity in extracts from brown algae occurring in the Baltic Sea (Kiel Fjord). One of the species growing in the Kiel Fjord is *Saccharina latissima* (S.l.). Whereas there are hundreds of publications about the fucoidan of *Fucus vesiculosus* and some other species, the SP of S.l. (S.l.-SP) have as of yet only been described by the group of Usov, who isolated them from S.l. growing in the Atlantic Ocean [18], as well as in the White and Barents Seas [19]. In pharmacological studies, they displayed a promising activity profile [20,21,22]. Remarkably, in a comparative study of nine different fucoidans isolated from nine species of brown algae, the fucoidan from *Laminaria saccharina* (syn. *Saccharina latissima*) was the most potent in terms of anti-inflammatory, anticoagulant, antiadhesive and antiangiogenic activities [22].

The aim of the presented study was to isolate and characterize the S.l.-SP of S.l. from the Kiel Fjord, Baltic Sea, and to compare them with those of S.l. from the North Atlantic. The Baltic Sea crucially differs from the North Atlantic in two parameters, namely its lower salinity (about 15 psu (practical salinity units) *versus* about 35 psu) and its very small tidal amplitude. Further differences concern e.g., waves, sea current, availability of hard substrate, diversity of species and occurrence of epibionts. To investigate whether these features have any impact on the S.l.-SP, additionally S.l. from the Faroe Islands in the North Atlantic was used for extraction. Since previous investigations showed that the harvest time influenced yield and purity of the sulfated xylogalactans (D.s.-SP) extracted from the red alga *Delesseria sanguinea* (D.s.) [14], extractions were also performed with S.l. batches harvested at different months.

The obtained S.l.-SP batches and fractions were structurally analyzed and tested for two exemplary pharmacological effects, namely the inhibition of human polymorphonuclear neutrophil elastase and the anticoagulant activity. These activities were selected, as the applied fluorimetric elastase assay and the activated partial thromboplastin time (APTT) were simple and validated methods and thus useful for comparative screening [23,24].

## 2. Results

### 2.1. Extraction of Crude Sulfated Polysaccharides of Saccharina latissima (S.l.-SP) from Four S.l. Batches

In the run-up to this project, the most appropriate extraction and purification procedure for S.l. was established and standardized. By targeted modifications of the process, overall 28 S.l. extracts were produced and compared. The standardized procedure that was ultimately used led to S.l. extracts mainly consisting of sulfated polysaccharides (crude S.l.-SP). Since the applied standardized isolation procedure turned out to be still associated with certain variability of yield and composition of crude S.l.-SP, each S.l. batch harvested from the Baltic Sea was extracted six times and each S.l. batch collected from the Atlantic Ocean was extracted eight times, respectively.

#### 2.1.1. Composition of Crude S.l.-SP

The composition of the crude S.l.-SP according to elemental analysis revealed significant differences in dependence of both habitat and harvest time of S.l. (Table 1).

A-SP had significantly higher sulfate contents and accordingly higher degrees of sulfation (DS) than B-SP (mean DS: 0.42 and 0.49 *vs*. 0.28 and 0.31). In addition, the DS of the crude S.l.-SP was the higher the later the S.l. had been harvested. Whereas the period of only six weeks between harvesting of the two B-S.l. batches was associated with a non-significant increase from 0.28 to 0.31, the DS increase was significant for A05-SP (DS 0.42) and A09-SP (DS 0.49).

**Table 1:** Basic Characteristics of the Crude S.l.-SP Extracted from Four S.l. Batches ^a^.

S.l.-SP Batches	Glycans ^b^ (%)	Sulfate ^b^ (%)	Protein ^b^ (%)	DS ^c^	IC_50_ (Elastase) ^d^ (μg/mL)
**B05-SP**	72.7 ± 2.2	14.4 ± 2.8	12.8 ± 1.0	0.28 ± 0.06	0.89 ± 0.13
**B06-SP**	74.2 ± 3.3	15.5 ± 4.0	10.3 ± 1.5	0.31 ± 0.09	0.66 ± 0.12
**A05-SP**	66.4 ± 1.0	19.0 ± 2.1	14.6 ± 2.7	0.42 ± 0.04	0.51 ± 0.05
**A09-SP**	67.6 ± 2.5	22.6 ± 2.6	9.8 ± 1.9	0.49 ± 0.07	0.41 ± 0.06
B05 v. B06	n.s.	n.s.	*p =* 0.017	n.s.	*p =* 0.020
B05 v. A05	*p* < 0.001	*p =* 0.012	n.s.	*p =* 0.003	*p =* 0.010
B05 v. A09	*p =* 0.008	*p* < 0.001	*p =* 0.019	*p =* 0.002	*p* < 0.001
B06 v. A05	*p* < 0.001	n.s.	*p =* 0.005	*p =* 0.021	*p =* 0.041
B06 v. A09	*p =* 0.002	*p =* 0.003	n.s.	*p =* 0.002	*p* < 0.001
A05 v. A09	n.s.	*p =* 0.016.	*p =* 0.003	*p =* 0.040	*p =* 0.013

^a^ Data are presented as means ± SD of at least six crude S.l.-SP extracts obtained from each of the four S.l. batches; ^b^ The contents of glycans, sulfate (indicated as −SO_3_Na) and protein (*i.e*., nitrogen (%)) were calculated from elemental analysis, which was performed at least two times for each crude S.l.-SP extract; ^c^ DS, degree of sulfation, means −SO_3_Na residues per monosaccharide related to the content of total glycans; ^d^ IC_50_ values represent the concentrations for 50% inhibition of elastase activity. Each extract was tested in duplicate and the assay was repeated at least two times on different days.

The elastase inhibitory activity clearly correlated with the DS: A09-SP with the highest DS exhibited the best activity with an IC_50_ of 0.41 ± 0.06 μg/mL.

Contrary to the sulfate content, the protein content of crude S.l.-SP significantly decreased the later the S.l. was harvested, but this might be just the consequence of the increased sulfate and glycan contents. No clear dependence of the protein content on the habitat of S.l. was found.

#### 2.1.2. Monosaccharide Composition of Crude S.l.-SP

To get more information about the glycan part of the crude S.l.-SP, their monosaccharide composition was examined by acetylation analysis (Table 2).

**Table 2:** Composition of Neutral Monosaccharides of Crude S.l.-SP (mol%) ^a^.

Crude S.l.-SP Batches	l-fucose	d-galactose	d-xylose	d-mannose	d-rhamnose	d-glucose
**B05-SP**	45.2 ± 14.4	8.3 ± 2.5	5.4 ± 1.5	8.3 ± 2.5	1.1 ± 0.2	32.7 ± 17.4
**B06-SP**	42.9 ± 11.3	6.9 ± 4.3	4.6 ± 1.1	6.8 ± 0.4	1.6 ± 0.2	37.5 ± 11.5
**A05-SP**	62.8 ± 8.2	14.9 ± 9.9	8.0 ± 0.9	8.9 ± 2.0	1.8 ± 0.5	4.7 ± 1.7
**A09-SP**	63.9 ± 6.1	10.6 ± 3.9	7.1 ± 0.7	7.0 ± 1.6	1.3 ± 0.5	10.1 ± 4.9

**^a^** Data are represented as the mean ± SD of at least six crude S.l.-SP extracts obtained from each of the four S.l. batches, whereby the acetylation analysis of each extract was carried out twice.

With 43%–64%, fucose showed to be the main monosaccharide. Moreover, galactose, xylose, mannose as well as small amounts of rhamnose were detected. The most pronounced difference between the B-SP and A-SP concerned the glucose amount. In B-SP, it amounted to at least one-third of the monosaccharides, whereas the A-SP contained at most 15% glucose. Since the used isolation procedure did not prevent co-extraction of the reserve glucan laminarin, the glucose content was assumed to reflect mainly the laminarin content as was subsequently confirmed.

Besides neutral monosaccharides, the crude S.l.-SP contained about 10-uronic acids as determined with the Blumenkrantz assay. The content showed to be independent of habitat and harvest time of S.l. (B05-S.l. 10.83% ± 0.20%, B06-S.l. 10.38% ± 0.18%, A05-S.l. 10.84% ± 0.07%, A09-S.l. 8.63% ± 0.13%). The uronic acids have been identified as glucuronic acids, since the acetylation analysis after uronic acid reduction resulted in correspondingly increased contents of glucose (data not shown).

According to colorimetric assays, the crude S.l.-SP were neither substituted with acetyl [25] nor pyruvate [26] groups.

#### 2.1.3. Yields and Degree of Sulfation (DS) of Crude S.l.-SP Compared with Those of the Sulfated Fucose and Galactose Residues of S.l.-SP (FGSP) and Glucose Proportions

A-S.l. resulted in significantly higher yields based on dry mass (A05-SP 4.24% ± 0.95% and A09-SP 3.75% ± 0.44%) than B-S.l. (B05-SP 1.75% ± 0.01% and B06-SP 2.27% ± 0.29%) (Table 3), whereas the time of harvest had no significant influence. The group of Usov isolated S.l.-SP in a yield of 2.2% of the dry defatted algal biomass [18] so that the yield based on dry mass was somewhat lower. The yield was therefore rather similar to those obtained from B-S.l., although they used also S.l. from the North Atlantic Ocean (northwestern Scotland in the Ullapool area). However, the alga was already harvested in March and the isolation and purification procedure differed in some details.

**Table 3:** Yields (%, Related to Dry Mass of S.l.) and DS of Crude S.l.-SP as well as of their FGSP ^b^ and Glucose ^c^ Proportions.

S.l.-SP Batches	Yield (%) ^a^			DS ^a^	
	Crude S.l.-SP	FGSP ^b^	Glucose ^c^	Crude S.l.-SP ^d^	FGSP ^e^
**B05-SP**	1.75 ± 0.01	0.94 ± 0.29	0.42 ± 0.00	0.28 ± 0.06	0.55 ± 0.11
**B06-SP**	2.27 ± 0.29	1.16 ± 0.31	0.63 ± 0.08	0.31 ± 0.09	0.60 ± 0.05
**A05-SP**	4.24 ± 0.95	3.05 ± 0.79	0.13 ± 0.04	0.42 ± 0.04	0.53 ± 0.04
**A09-SP**	3.75 ± 0.44	2.66 ± 0.10	0.26 ± 0.03	0.49 ± 0.07	0.67 ± 0.10
B05 v. B06	n.s.	n.s.	*p =* 0.037	n.s.	n.s.
B05 v. A05	*p =* 0.025	*p =* 0.025	*p =* 0.002	*p =* 0.003	n.s.
B05 v. A09	*p =* 0.004	*p* < 0.001	*p =* 0.002	*p =* 0.002	n.s.
B06 v. A05	*p =* 0.019	*p =* 0.012	*p* < 0.001	*p =* 0.021	n.s.
B06 v. A09	*p =* 0.004	*p* < 0.001	*p* < 0.001	*p =* 0.002	n.s.
A05 v. A09	n.s.	n.s.	*p =* 0.004	*p =* 0.040	*p =* 0.011

^a^ Data are presented as the mean ± SD of at least six crude S.l.-SP extracts obtained from each of the four S.l. batches; ^b^ Sum of the yields (%) of fucose, galactose and sodium sulfate; ^c^ Yield (%) of glucose; ^d^ DS calculated as −SO_3_Na residues per monosaccharide related to the content of total glycans; ^e^ DS calculated as −SO_3_Na residues per monosaccharide related to the content of fucose and galactose.

Fractionation and further analyses of the crude S.l.-SP revealed that almost all sulfate groups were bound to fucose and galactose residues so that the corresponding polymers (FGSP) were supposed to represent the bioactive components of crude S.l.-SP. Therefore, the yields (%, related to dry mass) were recalculated for the FGSP proportion and additionally for the glucose proportion as a rough indicator for laminarin.

As shown in Table 3, the FGSP yields from A-S.l. were about three times higher than those from B-S.l., whereas those of glucose were about three times lower.

Since the co-extracted laminarin is unsulfated, it can be considered as a “diluent” of the FGSP proportion and the charge density of the sulfated polymers is higher than reflected by the DS of the crude S.l.-SP. This was confirmed by the DS calculated for the respective FGSP proportions being up to twice as high as those of the crude S.l.-SP (Table 3). Whereas the DS of the crude B-SP batches was significantly lower than those of the A-SP, the DS of the corresponding FGSP were similar and thus independent of the habitat of S.l. (Supplementary Figure S1). However there was a dependence on the harvest time as indicated by the significantly higher DS of the FGSP from S.l. harvested in September compared to S.l. in May (0.67 ± 0.10 *vs*. 0.53 ± 0.04).

#### 2.1.4. Variability of Dry Mass of S.l. and Its Impact on the Yields

The peculiar result that the yield (%) related to dry mass of the high-sulfated FGSP of A09-SP was lower than that of A05-SP (Table 3) requested further consideration. Our previous study on the sulfated xylogalactans extracted from *Delesseria sanguinea* showed that the seasonal variability of the yields mainly resulted from differences of the dry mass % [14]. There the dry mass increase between April and October was attributed to an accumulation of the reserve compound floridean starch so that the yields related to dry mass decreased.

**Figure 1:**
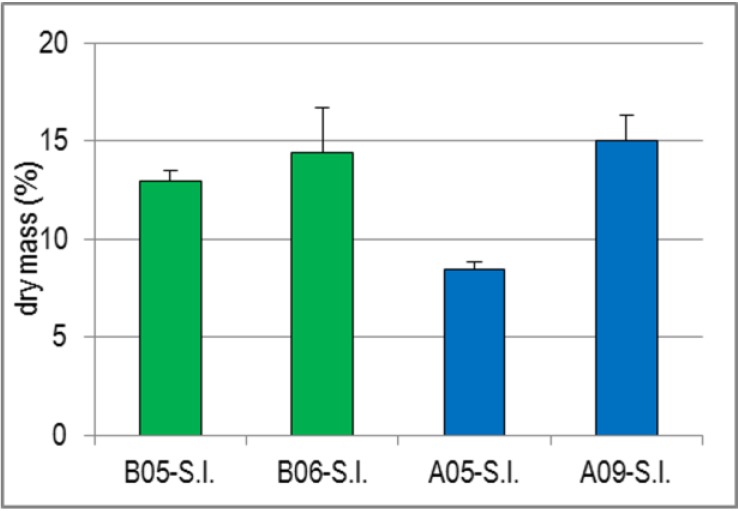
Dry Mass (%) of the Drained Fresh S.l. Fronds. Values represent the mean ± SD (*n* = 3), whereby the repeated measurements were performed with different net weights.

Similar to the findings with D.s., the dry mass determination of the four S.l. batches revealed a clear seasonal dependence, but additionally a striking difference between B-S.l. and A-S.l. (Figure 1). The dry mass of B-S.l. increased from 13.0% to 14.4% over a period of only six weeks, that of A-S.l. from 8.5% (harvested in May) to 15.1% (harvested in September). Since the dry mass of A09-S.l. was, however, higher than that of the two laminarin-rich B-S.l. batches without a corresponding increase of laminarin in A09-SP, the dry mass increase could not only result from an increase of reserve polysaccharides. Analogously, the higher dry mass of B05-S.l. compared to A05-S.l. cannot be explained by compounds contained in crude S.l.-SP, but is rather due to other constituents like alginic acid, mannitol and/or minerals [27].

Due to the dry mass differences, it was indicated to additionally compare the yields obtained from the extraction of 32.5 g drained fresh S.l. fronds each. For any commercial production of SP from S.l. it would be important to know what algae mass is needed for the production of a certain amount of SP.

**Figure 2:**
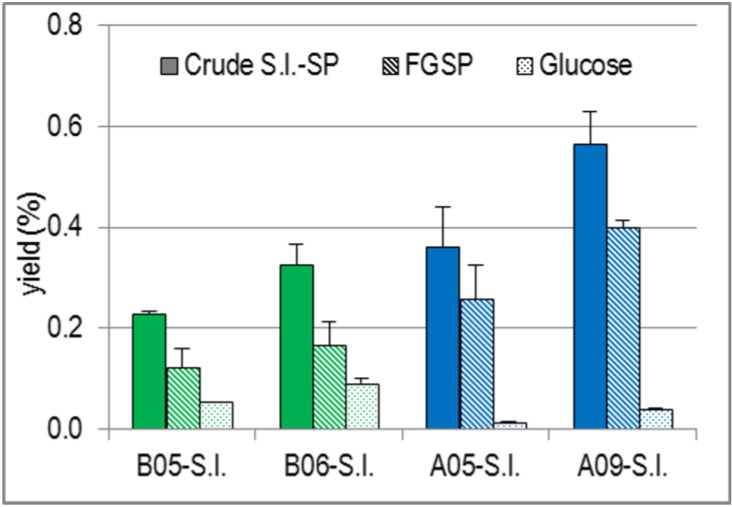
Yields (%) of Crude S.l.-SP as well as of Their FGSP and Glucose Proportions Related to 32.5 g Drained Fresh S.l. Fronds.

Regarding the yields related to drained fresh mass (Figure 2), A09-S.l. turned out to be the optimal S.l. batch. It yielded the highest amount of FGSP. To isolate the same amount of FGSP, a B05-S.l. mass of approximately three times higher mass had to be extracted. Comparing the two S.l. batches harvested in May, the yield of FGSP from B05-S.l. was only half as high than that from A05-S.l. During the summer period, the yield of FGSP from A-S.l. increased by about 60%.

The yield of glucose as an estimate for laminarin also showed a clear dependence on both the habitat and harvest time of S.l. Like FGSP, it increased during the summer period, but contrary to FGSP, B-S.l. was superior to A-S.l. From B05-S.l., five times more glucose was isolated than from A05-S.l. and the amount from B06-S.l., harvested six weeks later, was still 2.5 times higher than that from A09-S.l. harvested in September.

In summary, the comparison of the yields and the overall composition of the crude S.l.-SP from the four different S.l. batches revealed that the extractable amounts of both glucose/laminarin and the bioactive FGSP proportion increased during the summer months and that S.l. from the North Atlantic yielded significantly more FGSP, but less glucose/laminarin than S.l. from the Baltic Sea.

### 2.2. Fractionation of Crude S.l.-SP

**Figure 3:**
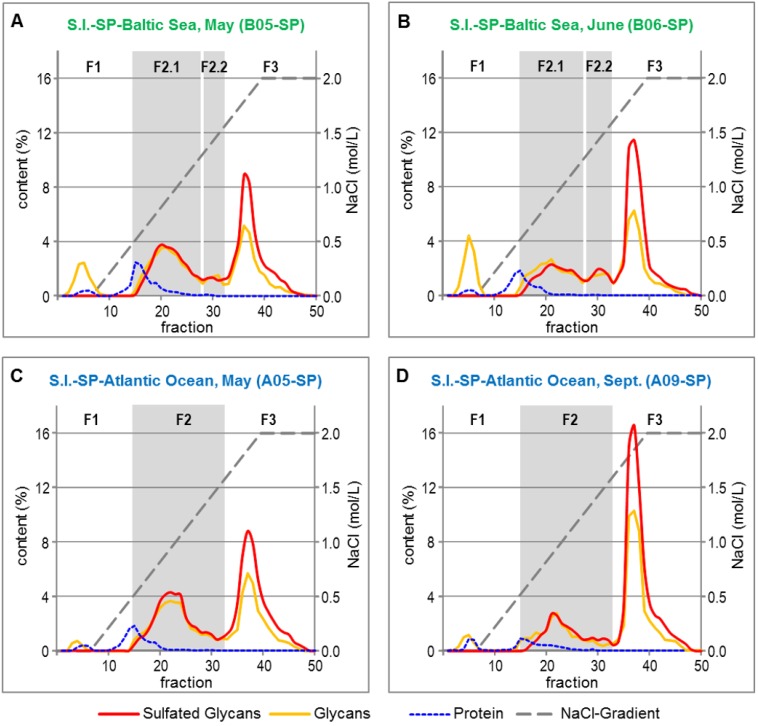
Fractionation of Crude S.l.-SP Batches by Anion Exchange Chromatography. SP from S.l. harvested from the Baltic Sea in May (**A**) and June (**B**), SP from S.l. harvested from the Atlantic Ocean in May (**C**) and September (**D**).

The crude S.l.-SP batches were fractionated by anion exchange chromatography (AEC) to elucidate the composition and quantitative distribution of the sulfated and non-sulfated compounds as well as to potentially eliminate proteins and any other co-extracted compounds. By means of the contents of glycans and sulfated glycans, four (B-SP) or three (A-SP), respectively, major fractions were identified (Figure 3). According to the corresponding chromatograms, the 4 mL fractions 1–14 (eluted at 0–0.54 mol/L NaCl), 15–32 (eluted at 0.55–1.63 mol/L NaCl) and 33–50 (eluted at 1.64–2 mol/L NaCl) were pooled and further analyzed.

The F1 fractions consisted of unsulfated glycan, 10%–26% of the overall protein content of the crude S.l.-SP batches and brownish colored compounds (Table 4). The latter are assumed to be water-extractable representatives of the complex group of phlorotannins, which are typical compounds of the cell walls of brown algae [28,29]. As found for the overall glucose content of the crude S.l.-SP batches (Table 2), the portion of unsulfated glycan was both dependent on the habitat and the harvest time of S.l.

The glycans of the F2 and F3 fractions were sulfated, whereby the courses of the anthron assay and DMMB assay curves suggested that those of F3 had a higher DS. The F3 fractions eluted as sharp peaks, whereas the sulfated glycans of the F2 fractions may be composed of a rather heterogeneous mixture of sulfated glycans. Additionally, the F2 fractions contained 74%–90% of the overall proteins of the crude S.l.-SP batches.

**Table 4:** Yields and Structural Composition of the S.l.-SP Fractions Obtained by AEC.

S.l.-SP	Yield (%) ^1^	Glycans (%) ^2,3^	Sulfate (%) ^2,4^	Protein (%) ^2,5^	Sulfated Glycans (%) ^6^	Monosaccharide Composition (mol %) ^7^					
						Fuc	Gal	Xyl	Man		Glc
**B05-SP**											
**F1_1–14_**	9.8	8.5	-	1.3	-	-	-	-	2.1		97.9
**F2.1_15–27_**	50.3	30.1	5.7	12	30.0	54.7	10.4	13.7	16.2		3.3
**F2.1_28–32_**		5.9		-	6.2						
**F3_33–50_**	39.9	25.5	13.8	-	42.0	67.0	26.5	2.8	2.4		1.3
		100% ^8^									
**B06-SP**											
**F1_1–14_**	12.1	11.5	-	1.1	-	-	-	-	2.7		97.3
**F2.1_15–26_**	41.7	21.4	5.7	8.9	18.7	54.3	13.7	13.5	12.7		3.4
**F2.2_27–32_**		8.2		-	9.2						
**F3_33–50_**	46.2	28.4	16.1	-	50.7	75.6	19.6	3.4	1.4		-
		100% ^8^									
**A05-SP**											
**F1_1–14_**	3.4	1.9	-	1.0	-						
**F2_15–32_**	50.5	36.1	5.5	8.9	38,8	46.9	11.2	15.3	16.0		8.4
**F3_33–50_**	46.1	29.5	15.7	-	49.5	63.4	31.8	2.6	1.4		0.9
		100% ^8^									
**A09-SP**											
**F1_1–14_**	5.1	3.2	-	1.9	-						
**F2_15–32_**	27.6	20.6	2.8	5.5	21.3	39.7	22.5	18.4	12.2		4.7
**F3_33–50_**	67.3	42.9	22.8	-	68.3	86.1	11.1	2.8	-		-
		100% ^8^									

^1^ Yield (%) in relation to crude S.l.-SP; ^2^ The contents (%) of glycan, sulfate and protein were determined for each fraction and then adjusted according to its respective proportion (yield (%)) of the crude S.l.-SP; ^3^ The glycan content (%) was determined by anthron assay and corresponds to the respective proportion of the area under the curve (AUC). The anthron assay results were in line with the glycan contents calculated by means of elemental analysis; ^4^ Sulfate content (%) calculated as −SO_3_Na from the sulfur content determined by elemental analysis; ^5^ Protein content (%) calculated from the nitrogen content determined by elemental analysis; ^6^ The sulfated glycan content (%) was quantified by DMMB assay and corresponds to the respective proportion of the area under the curve (AUC); ^7^ The neutral monosaccharide composition was examined by acetylation analysis. Fractions F2 and F2.1/F2.2 additionally contained small amounts of rhamnose (not indicated); ^8^ The addition of glycan, sulfate and protein contents of fractions F1−F3 resulted in about 100% S.l.-SP. Furthermore, the sum of sulfated glycan contents (F2 and F3), protein contents (F1 and F2) and glycan content of F1 (*i.e*., laminarin content) amounted to about 100% as well.

#### 2.2.1. Common Structural Characteristics of the S.l.-SP Fractions

In both B05-SP and A05-SP, F2 represented the main fraction with about 50%, whereas in B06-SP and A09-SP, the proportion of F3 was the highest (Table 4).

F2 Fractions. The F2 fractions were relatively low-sulfated (DS 0.19–0.28), but according to the Blumenkrantz assay and comparative acetylation analyses the charge density of all the F2 fractions was increased by uronic acids (B05-SP-F2 15.1%, B06-SP-F2 17.6%, A05-SP-F2 15.1% and A09-SP-F2 22%). In contrast to F2, all the F3 fractions had higher DS (0.76–0.82), but did not contain any uronic acids. The uronic acids in F2 were present as 3-linked, 4-linked and terminal unsulfated glucuronic acids as proven by methylation analysis after reduction with sodium borodeuteride.

The main monosaccharide of F2 showed to be fucose (about 40%–55%) followed by mannose, xylose, galactose, glucose and slight amounts of rhamnose. According to methylation analysis, mainly the galactose was sulfated. Both the elution profiles obtained by AEC (Figure 3) and the distinct variations of the monosaccharide compositions in dependence of the harvest time (e.g., in B06-SP and A09-SP increase of galactose, decrease of mannose) suggest that F2 was composed of a mixture of various heteropolysaccharides. This was further supported by SEC resulting in chromatograms with various peaks. For example, the chromatogram of A09-SP-F2 revealed to be constituted of at least three fractions with mean M_r_ of 453,000 ± 55,000, 221,000 ± 27,000 and 77,000 ± 10,000 (MALLS), respectively. Since the F2 fractions displayed only moderate elastase inhibitory activities (Table 5), no further fractionation was performed.

**Table 5:** Key Structural Characteristics and Activities of the S.l.-SP Fractions Obtained by AEC.

	Degree of Sulfation (DS)		Fucose/Galactose Ratio		IC_50_ (Elastase) (μg/mL) ^a^		DC (APTT) (μg/mL) ^b^
Fraction	F2	F3	F2	F3	F2 *	F3 *	F3 *
**B05-SP**	0.23	0.79	5.3	2.5	2.41 ± 0.12	n.d.	n.d.
**B06-SP**	0.28	0.82	4.0	3.9	1.87 ± 0.12	0.26 ± 0.02	7.44 ± 0.18
**A05-SP**	0.22	0.76	4.2	2.0	2.81 ± 0.21	0.28 ± 0.01	8.75 ± 0.25
**A09-SP**	0.19	0.81	1.8	7.8	3.77 ± 0.16	0.21 ± 0.01	4.89 ± 0.50

Reference UFH: IC_50_ (Elastase) 0.29 ± 0.02 μg/mL, DC (APTT) 1.10 ± 0.08 μg/mL; * The differences between the mean values are statistically significant (*p* < 0.05); ^a^ IC_50_ values represent the concentrations for 50% inhibition of elastase activity. Each batch was tested at least four times on different days (each duplicate determination); ^b^ DC values represent the inhibitor concentration in human pooled plasma prolonging the coagulation time to twice the time of the negative control. Each batch was tested at least three times on different days (each duplicate determination).

F3 Fractions. In contrast to F2, the composition of the high-sulfated F3 fractions (DS 0.76−0.82) was more homogeneous. In addition, the SEC of A09-SP-F3 revealed a single peak with a mean M_r_ of 416,000 ± 28,000 (MALLS) and 449,000 ± 15,000 (RI), respectively (Supplementary Figure S2). They consisted of more than 90% fucose and galactose and were free of proteins and uronic acids.

F1 Fractions. The glycan part of the smallest fractions F1 (3.4%–9.8%) was mainly composed of uncharged glucan containing some mannitol residues. By methylation analysis it was identified as laminarin, *i.e.*, 1,3-linked glucan with some branchings at C6.

#### 2.2.2. Composition of the SP Fractions Related to the Harvest Time of S.l.

To identify any impact of the harvest time of S.l. on its sulfated polysaccharides, the fractions of A05-SP and A09-SP were compared (Table 4). B05-SP and B06-SP with a period of 1.5 months between the times of harvest provided Supplementary Information.

(1) There was a clear harvest-time-dependent shift of the proportions of the three fractions. Whereas the amounts of lower-charged F2 decreased, those of F3 increased. In A09-SP, F3 amounted to 67.3% and the proportion of F2 (27.6%) was nearly halved compared to A05-SP. Furthermore, the F1 amounts also increased. A corresponding but smaller shift of the proportions was also observed for the fractions of B05-SP and B06-SP.

(2) The DS of A-SP-F3 increased from 0.76 to 0.81, whereas that of A-SP-F2 decreased from 0.22 to 0.19 (Table 5). This divergence of DS changes was not observed in B-SP; here, the DS of both F2 (0.23 *vs.* 0.28) and F3 (0.79 *vs*. 0.82) increased. However, this fits to the observation of the some higher sulfated F2.2 and its increase from 6.2% in B05-SP to 9.2% in B06.-SP.

(3) The harvest-time-dependent reduction of the yield (%) of F2 fractions was associated with a corresponding decrease of the percentage of protein in F2 related to the overall composition of the three fractions.

(4) The monosaccharide composition of F3 shifted to increased contents of fucose, whereas the proportions of both galactose and the other monosaccharides decreased. In A09-SP-F3, the latter were limited to 2.8% xylose. Inverse changes were observed in F2, *i.e*., decrease of fucose and increase of galactose.

(5) Consequently, the fucose/galactose ratios of F2 and F3 inversely changed (Table 5): There was a marked increase of the fucose/galactose ratio of F3 from 2.5 and 2.0, respectively, in the May batches over 3.9 in B06-SP to 7.8 in A09-SP, whereas that of F2 decreased from 5.3 and 4.2, respectively, over 4.0 to 1.8. This suggests that primarily glycans mainly consisting of fucose have been subjected to sulfation.

#### 2.2.3. Composition of the SP Fractions Related to the Two S.l. Habitats Baltic Sea and Atlantic Ocean

Although primarily the fractions of the two S.l.-SP batches isolated from algae harvested in May are suited for evaluating any influence of the habitat of S.l. on its extractable SP, additionally some harvest-time-independent differences were recognized (Table 4).

(1) As expected from the data of the crude S.l.-SP, the proportion of F1 (*i.e*., mainly laminarin) was nearly three times higher in B-SP than in A-SP.

(2) Moreover, the protein content of B-SP-F2 was considerably higher than that of A-SP-F2, whereby the protein was exclusively found in F2.1.

(3) In contrast to A-SP-F2, B-SP-F2 consisted of a larger fraction F2.1 and a smaller fraction F2.2 with some higher charge density. The latter was probably the reason for the overall higher DS of B-SP-F2 compared to A-SP-F2.

(4) B-SP-F2 contained significantly more fucose and had higher fucose/galactose ratios than A-SP-F2 (Table 5).

(5) In A-SP, however, the percentage of the high-fucose fraction F3 in relation to the yield of F2 + F3 was higher than in B-SP (A05-SP: 48% *vs.* B05-SP: 44%; A09-SP: 71% *vs*. B06-SP: 53%).

The presence of F2.2. in B-SP seems to compensate the higher percentage of F3 in A-SP and provides an explanation why the calculated DS of the FGSP proportions did not significantly differ between A-SP and B-SP (Table 3 and Supplementary Figure S1).

#### 2.2.4. Elastase Inhibitory and Anticoagulant Activities of the S.l.-SP Fractions

To investigate the impact of the chemical differences between the various S.l.-SP fractions on their pharmacological potency, they were tested in the chromogenic elastase activity assay (Table 5). Compared to crude S.l.-SP, the activities of the high-sulfated F3 fractions were improved by at least factor 2 (Table 1 and Table 5). Their IC_50_ were about one order of magnitude lower than those of F2. The most active fraction A09-SP-F3 had an IC_50_ even lower than that of the reference unfractionated heparin (0.21 ± 0.01 μg/mL *vs*. 0.29 ± 0.02 μg/mL). Whereas the crude B-SP, which contained considerably more laminarin, were about 60%–75% less active than the crude A-SP batches (Table 1), the activity differences between the corresponding F3 fractions were smaller and the F2 fractions of B-SP were even more active than those of A-SP.

As previously found for semisynthetic glucan sulfates [30], there was a clear correlation between the inhibitory activity and the DS of the various F2 and F3 fractions (*r* = 0.9982). Due to this DS dependence, the harvest time of S.l. indirectly influenced also the activity of S.l.-SP. Since the sulfation of the FGSP obviously continued during the summer months, the isolated F3 fractions had a higher DS (A09-SP-F3: 0.81 *vs.* A05-SP-F3: 0.76) and that was associated with improved activity (IC_50_ A09-SP-F3: 0.21 ± 0.01 μg/mL *vs*. IC_50_ A05-SP-F3: 0.28 ± 0.01 μg/mL). In contrast, the habitat of S.l. showed to be less important for the activities of the purified S.l.-SP fractions.

For comparison with published data, the S.l.-SP fractions were additionally tested in the global coagulation assay APTT (Table 5). In contrast to their elastase inhibitory potency, the anticoagulant activity of the F3 fractions amounted to only 12.6%–22.5% that of UFH (DC = 1.10 ± 0.08 μg/mL) and the F2 fractions (up to 1000 μg/mL) did not prolong the coagulation time at all. The activities of F3 fractions were 70% higher than those of the crude S.l.-SP, and the activity ranking of the various F3 fractions in the APTT was the same as found in the elastase assay. The anticoagulant activity of the F3 fraction of A09-SP was similar to that of the 1.25 mol/L NaCl fraction of North Atlantic-S.l. extract reported by Croci *et al.* [20], which exhibited 20.9% of the activity of UFH.

### 2.3. Structural Characterization of Fraction F3 from September

The fraction with the best elastase inhibitory activity and thus the most interesting one for further pharmacological investigations was F3 from A09-SP, *i.e*., the extract from S.l. harvested at the Faroe Islands in September. Moreover, among all the S.l.-SP batches, A09-SP was that with the highest overall yield (Figure 2), as well as that with the highest portion of the high-sulfated fraction F3 (67.3%). To further characterize A09-SP-F3, it was subjected to glycosidic linkage analysis by GLC-MS analysis (methylation analysis).

The applied methylation analysis procedure allows the analysis of sulfated glycans, but no distinction between glycosidically linked and sulfated hydroxyl groups. To get information about the position of the sulfate groups, the methylation data of native A09-SP-F3 were compared with those of desulfated A09-SP. A limitation of this strategy was that all the applied solvolytic desulfation procedures either resulted in incomplete desulfation or marked degradation of the SP from S.l. In case of A09-SP-F3, degradation during desulfation led to considerably loss of fucose as also described by Usov [31].

Methylation analysis of native A09-SP-F3 yielded 83.1% fucopyranose and 16.6% galactopyranose (Supplementary Table S1). Taking account of the methylation data of desulfated A09-SP-F3 as well as published results of methylation analysis and NMR spectroscopy [18], the methylation data support a 3-linked fucopyranose chain with about one C-2-branched fucose per seven fucose residues (degree of branching (DB) = 5.98) as main structural element (Table 6). The fucose showed to be either sulfated at C-4 (35.3% and 8.5%) or at C-2 (15.5%). The amount of 23.8% 2-substitued fucose is assumed to be represent 9.3% C-2-sulfated terminal fucose (besides 1.5% terminal galactose-3,6-disulfate) according to the overall 10.8% branched fucose (8.5%) and galactose (2.3%) residues and 14.5% 2-linked fucose. The high amount of terminal fucose found in desulfated A09-SP-F3 (Supplementary Table S1) is assumed to result partly from degradation of the fucan chain during desulfation. Consistent with Bilan *et al.* [18], a major part of galactose (7.6%) was 6-linked and sulfated at C-3, whereby about one-third (2.3%) was additionally branched at C-4. Furthermore, unsulfated 3-linked, C-3-sulfated 4-linked and C-3/C-6-bisulfated terminal galactose residues were identified. These interpretations resulted in 81.7% sulfate groups bound to fucose and galactose, respectively, which matched well with the DS of A09-SP-F3 (0.81) according to elemental analysis.

**Table 6:** Proposed Structural Composition of A09-SP-F3 Deduced from Methylation Analysis.

mol %	Glycosidic Linkage	Position of Sulfate	Sulfate (mol %)
**83.1**	**Fucose**		
9.3	Fuc*p*-(1→	2	9.3
14.5	→2)-Fuc*p*-(1→	-	-
35.3	→3)-Fuc*p*-(1→	4	35.3
15.5	→3)-Fuc*p*-(1→	2	15.5
8.5	→2,3)-Fuc*p*-(1→	4	8.5
**16.6**	**Galactose**		
1.5	Gal*p*-(1→	3 + 6	3.0
5.0	→3)-Gal*p*-(1→	-	-
2.5	→4)-Gal*p*-(1→	3	2.5
5.3	→6)-Gal*p*-(1→	3	5.3
2.3	→4,6)-Gal*p*-(1→	3	2.3
			81.7

As a further monosaccharide only small amounts of 4- or 2-linked and terminal xylose were detected in the desulfated sample (probably quantifiable due to the loss of fucose), whereas the recovery of xylose after methylation of the native sample was even lower (Supplementary Table S1). Nevertheless, its exclusive detection in the lipophilic phase (in contrast to sulfated glycans, which are found in the hydrophilic phase) suggests the presence of a small amount of unsulfated xylan. This agrees with the conclusions of Bilan *et al.* [18] from NMR spectroscopy that there were separate 1,4-linked β-xylan chains, although xylans are unknown to be present in brown algae.

In summary, the structural analysis of A09-SP-F3 indicated a sulfated galactofucan (SGF) with a homogeneous M_r_ profile (mean M_r_ 420,000) and a DS of about 0.8. Thus, SGF represents a high-sulfated subfraction of the FGSP. Chains of 3-linked fucopyranose residues, either C4- or C2-sulfated and with about one C-2-branched fucose per seven fucose residues were identified as the major structural component. Further components were sulfated terminal and 2-linked fucose, as well as 16.6% mainly C-3-sulfated galactose, whereby about one galactose (*i.e*., 1,4,6 Gal) per 6−7 galactose residues was branched.

## 3. Discussion

According to AEC and further analysis, the crude S.l.-SP were composed of a small fraction of laminarin, some proteins and phlorotannins (F1), a homogeneous high-sulfated fraction (F3), which was identified as sulfated galactofucan (SGF) with a DS of about 0.8 and a mixture of lower sulfated, but uronic acid and protein containing SP (F2). The presence of proteins in purified fucoidan fractions was observed in numerous studies [17]. Recent investigations indicated that these proteins are tightly associated with certain fucose-containing SP similar to proteoglycans and are likely to be of structural importance [17]. This assumption was corroborated by our observation that the harvest-time-dependent decrease of the percentage of F2 was associated with a corresponding decrease of protein.

Overall, the separation of the crude S.l.-SP into three fractions was comparable to that reported by Bilan *et al*. [18]. The finding that F3 of A09-SP (*i.e*., SGF) was more homogeneous than the main S.l. extract fraction eluted with 1.25 mol/L NaCl from Bilan *et al.* could be due to S.l. material from a different habitat and/or due to the use of Q Sepharose fast flow®, a stronger anion exchanger than DEAE-Sephacel, and linear gradient elution instead of step elution. The data on elastase inhibition as an exemplary activity demonstrated that the applied fractionation procedure is not only appropriate to produce purer and more homogeneous S.l.-SP, but also leads to compounds with considerably improved activity.

### 3.1. Increase of Laminarin as well as FGSP and Sulfated Galactofucan in S.l. from Spring to Autumn

The harvest-time-dependent increase of the glucose / laminarin content in crude S.l.-SP (Figure 2 and Table 4) is in line with previous studies including the afore mentioned study on the seasonal variations of the D.s.-SP from the red alga D.s. [14,27,32,33]. In macroalgae, reserve carbohydrates accumulate during the summer months and serve as food reserve substances in the winter months, when photosynthesis is reduced. Also the harvest-time-dependent increase of the dry mass of S.l. (Figure 1) and of the yields of crude S.l.-SP and FGSP (*i.e*., polymers consisting of fucose, galactose and sulfate, used as an estimate of bioactive S.l.-SP) (Figure 2) agrees with the results of Black [27,32]. He described that the dry mass of Laminariaceae, as well as their fucoidan content varied parallel to laminarin and mannitol. Consequently, the cell walls of the S.l. fronds changed from spring to autumn by incorporating especially the negatively charged FGSP. In addition, the FGSP proportion of the A09-SP batch had a higher DS than that of A05-SP (Table 3) so that the cell walls became considerably stronger negatively charged.

More detailed information was obtained by the results of the fractionation of the crude S.l.-SP including a harvest-time-dependent increase of the percentage of the high-sulfated F3 fractions and changed compositions of the fractions with a strong increase of the proportion of fucose (Table 4). Accordingly, further SGF (F3) was produced during the summer so that it became the main fraction of the S.l.-SP from A-S.l. In addition, the DS increase found for the crude S.l.-SP, the FGSP as well as F3 of the S.l.-SP primarily concerned the SGF. The latter confirms the supposed sequential biosynthesis of sulfated fucans: By analogy with the biosynthesis of glycosaminoglycan in vertebrates, sulfated fucans are likely to be polymerized as neutral polysaccharides by one or several fucosyltransferases and then sulfated by specific sulfotransferases [28]. Such a chronology is supported by the M_r_ profile of the S.l.-SP fractions: The higher sulfated fractions F3 consisted of a homogeneous fraction with high M_r_, whereas the fractions F2 displayed a heterogeneous M_r_ profile and contained considerable amounts of smaller polymers. This corresponds to the recently reported M_r_ profile of the SP from various brown algae, which switched from the 0.5 mol/L to the 2 mol/L NaCl AEC fractions towards large polymers [17].

All these alterations may be generally concomitant of growing S.l. fronds, which is most pronounced in summer, when intense photosynthesis supplies the required energy. Accordingly, the more energy consumed for production of FGSP and especially SGF, the less energy is left for formation und storage of laminarin. This may explain why A-S.l. containing more FGSP yielded less laminarin than B-S.l.

### 3.2. Inverse Yields of FGSP/Sulfated Galactofucan and Laminarin from Baltic Sea and Atlantic Ocean S.l. Batches

Compared to B-S.l., extraction of A-S.l. delivered higher yields of crude S.l.-SP and the crude S.l.-SP contained more FGSP (Table 3) and SGF (Table 4), respectively. The question arises as to why already in May fronds of A-S.l. in their first year growth contained more FGSP than those of B-S.l. Though the knowledge of the physiological functions of fucose-containing sulfated polysaccharides and the regulation of their biosynthesis is still limited, they appear to play a role in the algal cell wall organization and are thought to confer adaptive advantages, e.g., via osmotic or structural functions in saline and intertidal environments [5,15,16,17,28,34,35,36]. Current investigations proved that a portion of the FCSP is tightly associated with cellulose microfibrils and thus act as major cross-linking glycans in brown algal cell walls [17].

It is known that numerous environmental parameters like water temperature, light intensity and exposure, salinity, dissolved CO_2_, nutrients, intertidal amplitude, water current and biotic interactions impact the biosynthetic processes and thus the chemical composition of macroalgae [37,38]. For S.l. harvested from the Artic as well as from the North Sea it was shown that the chemical components were significantly affected by temperature and less by the partial pressure of CO_2_ [39]. However, any effects on the SP were not investigated.

Thus far, no specific examinations of the environmental factor-dependent amount and composition of SP have been published. Regarding the two habitats of the S.l. batches investigated in this study, two striking differences concern the tidal amplitude and the salinity (Kiel Fjord about 15 psu *versus* Faroe Islands about 35 psu). It was therefore of interest to check whether these parameters could be at least partially responsible for our results.

Investigations on the occurrence and function of SP in plants revealed that both the concentration of SP and their DS were positively correlated with salinity [5]. The starting point for that study was the hypothesis of a possible association between SP and salt stress or salt resistance, as SP are ubiquitously present in marine algae, but have thus far only been found in halophytes. According to Aquino *et al.* [5], their results suggest that the presence of SP in plants is an adaptation to high salt environments, which may have been conserved during plant evolution from marine green algae. Another, but not contradictory explanation for the loss of sulfate substitution in plant matrix polysaccharides was given by Michel *et al*. [28]: A major environmental change during the evolutionary transition from marine to terrestrial environments was the lower salinity, including a dramatic decrease in the availability of sulfate ions required for the biosynthesis of SP.

The hypothesis of an association between SP and adaption to environments with high salinity and/or large tidal amplitudes was supported by experiments with an Ectocarpus strain isolated from a genuine freshwater environment and re-exposed to seawater [34]. Most recently, it was confirmed that sulfated fucans were overproduced by the freshwater strain of Echocarpus upon acclimation to 100% seawater [17].

Considering the current state of knowledge of associations between SP and saline as well as intertidal environments, it seems plausible that S.l. from the Baltic Sea has adapted to the lower salinity and the lower tidal amplitude of its living environment and therefore produces less SP. Since less SP are required to cope with salt stress and osmotic shocks at low tides, the energy for their production can be saved and allows the synthesis and storage of more reserve carbohydrates as found in the crude S.l.-SP from the Baltic Sea.

The assumption of environment-triggered differences between A-SP and B-SP was further substantiated by the results of their fractionation. Compared to A-SP, the proportion of the laminarin (F1) was larger in the B-SP, whereas that of the high sulfated SGF (F3) was smaller. Instead, B-SP contained a small intermediate-charged fraction (F2.2) of (still) lower-sulfated FGSP and/or sulfated galactofucans. As indicated by the higher protein content in F2, the proportion of low sulfated proteoglycan-like SP was higher in B-SP than in A-SP.

Comparing the May batches, the SP in the cell walls of A-S.l. fronds were higher sulfated and consisted to a larger proportion of SGF than those of the B-S.l fronds, but the most striking difference was the higher amount of SP and thus the higher yield of FGSP obtained by extraction of A-S.l. (Figure 2). By additionally considering the composition of A09-SP, the following interpretation is supported: The production of SGF (F3) having the highest DS and largest M_r_ among the S.l.-SP was stimulated by the environment of the Faroe Islands, whereas this development process of S.l. fronds from the Baltic Sea in their first year of growth seemed to be retarded.

### 3.3. High DS and High Fucose Content as Determinants of Elastase Inhibitory Activity

Since the elastase inhibitory activity of a SP correlates with numerous other activities of SP relevant for antitumor and anti-inflammatory potency, it was used in this study to estimate the bioactivity of the S.l.-SP and their fractions. In terms of the objective to obtain S.l.-SP with best possible activities and in high yields, S.l. from the Faroe Island harvested in September proved to be the most suitable S.l. batch (A09-S.l.) (Table 1 and Figure 2). AEC of crude S.l.-SP led to the high-sulfated fractions F3 with considerably improved activities (Table 5). In general, there was an excellent correlation between the elastase inhibitory activity and the DS of the S.l.-SP batch or fraction.

The activity difference between A09-SP-F3 (DS 0.81, IC_50_ 0.21 ± 0.01 μg/mL) and B06-SP-F3 despite similar DS (DS 0.82, IC_50_ 0.26 ± 0.02 μg/mL) suggests that also parameters other than the DS influence the activity. The two fractions considerably differed in their fucose content (A09-SP-F3: 86.1%, B06-SP-F3: 75.6%) and their fucose/galactose ratio (A09-SP-F3: 7.8, B06-SP-F3: 3.9). The question arises whether a high content of fucose in sulfated glycans is beneficial for elastase inhibitory as well as anticoagulant activities.

Since fucose is a desoxymonosaccharide, its sulfate groups are exclusively bound to secondary hydroxyl groups. It is known that not only the DS but also the distribution of the sulfate groups is important for the activity of SP. For example, semisynthetic β-1,3-glucan sulfate with C2- and C4-bound sulfate groups exhibited stronger anticoagulant and elastase inhibitory activities than equally charged β-1,3-glucan sulfate with predominantly C6-bound sulfate groups [40]. For sulfated α-L-fucans from the egg jelly coat of the sea urchin, the sulfation pattern turned out to be an important feature for recognition of fucans by the sperm receptor contributing to the species-specificity of fertilization. The reactivity of the sperm did not correlate with the charge density of the fucan, but with the proportion of C2- and C4-sulfation [41]. However, hypotheses about a correlation between activity and either fucose itself or its sulfation pattern need comprehensive and systematic further investigations.

### 3.4. Sulfated Galactofucan as the Most Active Fraction of S.l.-SP

According to structure analysis, the most active fraction A09-SP-F3 is supposed to be a homogeneous sulfated galactofucan with a mean M_r_ of 420,000 and a DS of 0.81.

Despite some differences of the isolation and fractionation procedure, the structure of A09-SP-F3 turned out to be quite similar to 1.25 mol/L NaCl fraction of the extract from North Atlantic-S.l. harvested in March 2004 [18]. By comprehensive NMR spectroscopy both fucan and fucogalactan structures were identified, which are in line with our data. However, the question whether these glycans are covalently interconnected or not could not be clarified, especially since NMR spectroscopy was performed with the desulfated and therefore degraded fraction. Although our NMR data were not helpful, the M_r_ profile of A09-SP-F3 (*i.e*., a single peak) suggests rather a galactofucan than a mixture of fucan and fucogalactan structures.

## 4. Experimental

### 4.1. Algae Material

In 2011, fronds of different *Saccharina latissima* (Linnaeus) C.E. Lane, C. Mayes, Druehl and G.W. Saunders, Laminariaceae, batches were collected: Two batches growing in the Kiel Fjord, Baltic Sea, one harvested in May (B05-S.l.) and the other one six weeks later in June (B06-S.l.), as well as two batches of S.l. growing at the sublittoral habitat at the coastal region of the Faroe Islands in the North Atlantic Ocean, one harvested in May (A05-S.l.) and the other one in September (A09-S.l.) (unfortunately, algae material from the Baltic Sea harvested in September was not available). The kelp material from the Atlantic Ocean was harvested at low tide. The latter is also referred to as *Laminaria faeroensis* (Børgesen) Børgesen, which is, however, currently regarded as a taxonomic synonym of *Saccharina latissima* (Linnaeus) C.E. Lane, C. Mayes, Druehl and G.W. Saunders [42]. Fronds in their first year of growth with similar habitus were selected to exclude any age-related effects.

### 4.2. Extraction and Isolation

The dried algal biomass was ground and defatted by Soxhlet extraction with ethanol 99% (v/v) for 12 h. After evaporation of the ethanol, the material was extracted for 2 h with 2% aqueous calcium chloride under reflux conditions. By using aqueous calcium chloride as the extraction solvent, the alginates of the cell wall were transformed into insoluble, non-extractable calcium salts [43,44]. In general, a defined ratio of algae biomass to extracting agent volume of 37.5 g/L was used. The obtained raw extracts were centrifuged (10,000 *g*, 30 min, 4 °C) and the supernatants were concentrated 10-fold by evaporation. To precipitate the S.l.-SP, ice-cold 96% (v/v) ethanol was added resulting in a final ethanol concentration of 60% (v/v). After storing for three days at 4 °C followed by centrifugation (10,000 *g*, 30 min, 4 °C), the sediments were dissolved in demineralized water. Finally, the solutions were exhaustively dialyzed against flowing demineralized water at 6 °C (Spectra Por dialysis membranes, Spectrum, MWCO 1000, Carl Roth, Karlsruhe, Germany), adjusted to pH 7.0–7.4 with NaOH and freeze-dried to give crude S.l.-SP.

### 4.3. Fractionation

The crude S.l.-SP were fractionated by anion exchange chromatography (AEC). According to the manufacturer’s instructions, a Pharmacia XK 16/20 column (1.6 cm × 20.0 cm, Pharmacia, Uppsala, Sweden) was packed with Q Sepharose fast flow^®^ (GE Healthcare Life Sciences, Freiburg, Germany) (V_bed_ 30 mL), a strong anion exchanger with quaternary amine groups bound to a 6% cross-linked agarose, and equilibrated with sodium acetate buffer (0.05 mol/L, pH 6). The crude S.l.-SP samples (23 mg dissolved in 10 mL) were applied to the column and eluted with a linear NaCl gradient with a flow rate of 5 mL/min. After elution with the sodium acetate buffer for 6.4 min (incl. 2.8 min for V_dead_), the NaCl concentration of the sodium acetate buffer was continuously increased by 0.076 mol/L per minute up to 2 mol/L NaCl reached after 26.4 min. Increasing the molarity of NaCl to >2 mol/L did not result in elution of any further compounds. Starting after 2 min, 50 fractions (each 4 mL) were collected over a period of 40 min. After analyzing the fractions for their content of total carbohydrates (anthrone assay), sulfated glycans (DMMB assay) and protein (OPA assay), adequate fractions were pooled, dialyzed (MWCO 1000, Carl Roth, Karlsruhe, Germany) and lyophilized.

### 4.4. Analytical Testing of AEC Fractions

The analyses of the 50 fractions were performed in microplates (Nunc, Roskilde, Denmark).

#### 4.4.1. Sulfated Glycans

The concentration of sulfated glycans was determined by a modified version of the colorimetric dimethylmetylene blue assay (DMMB; dimethylmethylene blue hydrochloride, AppliChem, Darmstadt, Germany) [45].

#### 4.4.2. Total Carbohydrates

The concentration of total carbohydrates was determined by the colorimetric anthrone method (Anthron, Merck, Darmstadt, Germany) [46].

#### 4.4.3. Total Proteins

The total protein content was quantified by the fluorescence assay using ortho-phthalaldehyde according to the manufacturer’s instructions (Fluoraldehyde OPA Reagent Solution, Pierce, Rockford, Illinois, USA) [47,48].

### 4.5. Monosaccharide Composition by Acetylation Analysis

To determine the neutral monosaccharide composition, crude S.l.-SP and S.l.-SP fractions, respectively, were hydrolyzed with 2 mol/L trifluoroacetic acid (TFA) at 121 °C [49] and, after evaporation of TFA, converted into alditol acetate derivatives (AA) by reduction and acetylation [50]. The AA were separated by gas liquid chromatography (GLC) on an OPTIMA-225-0.25 μm fused silica capillary column (25 m × 0.25 mm i.d., film thickness 0.25 μm, Macherey-Nagel, Düren, Germany) using an HP 6890 gas chromatograph (Hewlett-Packard, Palo Alto, CA, USA) with integrated flame ionization detector. The helium flow rate was 1 mL/min, the oven temperature was 180 °C for 5 min followed by an increase of 1 °C/min up to 210 °C held for 10 min, the temperature of injector and detector was 250 °C and 240 °C, respectively. The AA were identified by their retention times. For quantitative analysis, the crude S.l.-SP samples were supplemented with a defined amount of myo-inositol as the internal standard. The percentage of the respective AA was calculated by applying the software HP GC Chemstation, Rev. A.06.03 (509) (Hewlett-Packard, Palo Alto, CA, USA).

To also detect the contained uronic acids, the samples were reduced before hydrolysis (see below) and transformed into AA.

### 4.6. Monosaccharide Linkages by Methylation Analysis

For glycosidic linkage analysis, both the native and the desulfated crude S.l.-SP and S.l.-SP fractions, respectively, were subjected to methylation analysis. The transformation of the desulfated samples into partial methylated alditol acetates (PMAA) was performed according to Harris *et al*. [51], whereas the native samples were first converted into the pyridinium salt form, then dried and methylated thrice. The partially methylated samples were isolated from the water phase by dialysis. After hydrolysis and desulfation by treatment with 2 mol/L TFA for 1 h at 121 °C, they were reduced with 0.5 mol/L sodium borohydride and finally acetylated with acetanhydride resulting in the corresponding PMAA. The PMAA were separated and detected by GLC-MS using a OPTIMA-1701-0.25 μm fused silica capillary column (25 m × 0.25 mm i.d., film thickness 0.25 μm, Macherey-Nagel, Düren, Germany) and a Hewlett-Packard 5890 Series II Gas Chromatograph (Hewlett-Packard, Palo Alto, CA, USA) coupled with a Hewlett-Packard MS Engine 5898A (electron ionization 70 eV, Hewlett-Packard, Palo Alto, CA, USA). The helium flow rate amounted to 1 mL/min. The temperature of the injector was 260 °C, the temperature program of the GLC oven consisted of the following phases: (1) 120 °C for 3 min; (2) increase by 8 °C/min up to 170 °C; (3) increase by 0.5 °C/min up to 185 °C; (4) increase by 20 °C/min up to 250 °C; and (5) 250 °C for 19.5 min. PMAA were identified by both their relative retention times of the total ion chromatogram and their mass spectra using a spectra library as well as the HP G 1034 C software for MS Chemstation (Hewlett-Packard, Palo Alto, CA, USA). For quantitative analysis, the samples were supplemented with a defined amount of myo-inositol as internal standard.

### 4.7. Chemical Modifications

#### 4.7.1. Desulfation

Among several tried methods, the most suitable for the S.l.-SP turned out to be the following solvolysis [52]: the native samples were transformed into the pyridinium salt form and then desulfated by incubation with dimethyl sulfoxide containing 10% (v/v) methanol for 7 h at 100 °C followed by dialysis and lyophilization.

#### 4.7.2. Reduction of Uronic Acids

The carboxyl groups of uronic acids were reduced to hydroxyl groups with sodium borodeuteride (98 atom% D; Sigma-Aldrich, St. Louis, MO, USA) according to Taylor and Conrad [53].

### 4.8. Molecular Mass (M_r_)

The M_r_ and the hydrodynamic volume (M_HV_) of crude S.l.-SP and S.l.-SP fractions were examined by size exclusion chromatography (SEC) with online multi-angle laser light scattering (MALLS), as well as refractive index (RI) detection using a PL‑GPC 50 Plus system with degasser and integrated differential RI detector (Polymer Laboratories, Varian Inc., Palo Alto, CA, USA) coupled with a miniDAWN MALLS detector (Wyatt Technologie Corporation, Dernbach, Germany). For the separation by size, a PL aquagel‑OH Guard 8 μm precolumn followed by three PL aquagel‑OH Mixed 8 μm (Agilent Technologies, Santa Clara, CA, USA) columns in series were used. The samples (2 mg/mL in elution buffer, injection volume 150 μL) were eluted with 0.1 M NaNO_3_ (pH 7, containing 0.05% NaN_3_) at a flow rate of 0.7 mL/min, the column temperature was kept at 35 °C by a column oven. The M_r_ values were calculated with ASTRA for Windows software version 4.70.07 (Wyatt Technologie Corporation, Dernbach, Germany). The M_HV_ was determined by calibration with pullulan standards (Polymer Laboratories, Varian Inc., Palo Alto, CA, USA).

### 4.9. Elemental Analysis

The contents of carbon, hydrogen, nitrogen and sulfur contents in crude S.l.-SP and S.l.-SP fractions were determined by elementary analysis performed with the HEKAtech CHNS Analyser (HEKAtech GmbH, Wegberg, Germany, calibrator: sulfanil amide). After gas chromatographic separation (carrier gas: helium), the respective analyte gases were detected in a thermal conductivity detector. Based on the sulfur content (%), the content of sulfate groups (calculated as −SO_3_Na) as well as the mean degree of sulfation (DS number of sulfate groups per monosaccharide) were calculated. The total protein content was estimated by multiplying the nitrogen content (%) by 6.25.

### 4.10. Uronic Acids

Uronic acids were quantified by reaction with m-hydroxydiphenyl (Fluka) according to the method by Blumenkrantz and Asboe-Hansen [54].

### 4.11. Pharmacological Methods

In the pharmacological assays, 0.9% NaCl instead of crude S.l.-SP and S.l.-SP fractions dissolved in 0.9% NaCl served as negative control. Unfractionated heparin (UFH) from porcine mucosal origin (200 IU/mg, Lot No 73508019, Novartis, Nürnberg, Germany) was used as reference compound.

#### 4.11.1. Elastase Inhibition Assay

The inhibitory activity of crude S.l.-SP and S.l.-SP fractions on elastase was investigated by a fluorigenic microplate assay using elastase from human polymorph nuclear granulocytes (EC 3.4.21.37, Merck Millipore, Darmstadt, Germany) and MeOSuc-Ala-Ala-Pro-Val-7-amido-4-methylcoumarin (Bachem, Bubendorf, Switzerland) as substrate as previously described [23,30]. By means of the concentration-dependent inhibition curves, the concentration for 50% inhibition of elastase activity (IC_50_ in μg/mL) was calculated.

#### 4.11.2. Activated Partial Thromboplastin-Time (APTT)

The anticoagulant activity of crude S.l.-SP and S.l.-SP fractions was examined by the APTT using Pathromtin SL (Siemens Healthcare Diagnostics, Eschborn, Germany) as previously described [24]. The coagulation time of pooled human platelet-poor plasma spiked with the samples dissolved in NaCl 0.9% was measured with an Amelung-coagulometer KC10 macro (Lemgo, Germany). By means of the concentration-dependent curves, the doubling concentration (DC in μg/mL) was determined, *i.e*., the inhibitor concentration causing a prolongation of the coagulation time to twice the time of the negative control (*i.e*., NaCl 0.9% instead of the sample).

### 4.12. Statistical Analysis

All analytical and pharmacological testing of the crude S.l.-SP and S.l.-SP fractions was performed in duplicate and repeated at least twice on different days. The data were analyzed for significant differences using Student’s *t*-test (Sigma Plot 11.0, Systat Software, Inc., San Jose, CA, USA), *p* ≤ 0.05 was considered statistically significant.

## 5. Conclusions

An important issue of the presented results is the influence of the chosen alga material on SP extracted from brown algae. In this study, the SP extracted from S.l. fronds in their first year of growth were shown to be composed of a homogeneous fraction of sulfated galactofucan (SGF) with a DS of about 0.8 and pronounced bioactivity and a heterogeneous mixture of low-sulfated, uronic acid and protein containing heteropolysaccharides.

The comparison of S.l.-SP obtained from S.l. batches harvested from the Baltic Sea and North Atlantic in May, June and September revealed marked differences of yield, composition and bioactivity of extractable S.l.-SP.

As supported by the current knowledge of the cell wall structure of brown algae and of the dynamic changes and evolutionary mechanisms involved in their adaptation to environmental conditions, the results indicated that high salinity and the stressors of intertidal sites are two important environmental parameters. Although other environmental factors such as temperature, light intensity and the availability of hard substrates impact the biosynthetic processes of S.l., overall the habitat conditions of the North Atlantic Ocean seemed to stimulate the production of S.l.-SP and especially of SGF as well as the incorporation of fucose in SGF and its substitution with sulfate groups so that a homogeneous, high-sulfated SGF becomes the main fraction among the S.l.-SP of one-year-old fronds in autumn. In contrast, this development process turned out to be retarded in S.l. growing in the Baltic Sea, which instead forms and stores more laminarin than the North Atlantic-S.l. during the growth period in summer.

Since the bioactivity of SGF proved to correlate with its DS and fucose content, habitat and harvest time of S.l. are relevant regarding any utilization of these SP. In conclusion, the results of this study corroborate that among the environmental factors influencing the development and chemical composition of algae, habitat and harvest time of brown algae should be considered as factors influencing the yield as well as the composition and thus the bioactivity of the fucose containing SP.

## Supplementary Files

Supplementary File 1

## Abbreviations

AEC: anion exchange chromatography
A-SP: sulfated polysaccharides from S.l. harvested from the Atlantic Ocean in May (A05-SP) or September (A09-SP)
B-SP: sulfated polysaccharides from S.l. harvested from the Baltic Sea in May (B05-SP) or June (B06-SP)
D.s.: Delesseria sanguinea
D.s.-SP: sulfated polysaccharides extracted from red alga Delesseria sanguinea
DS: degree of sulfation
FGSP: amount of sulfated fucose and galactose residues of S.l.-SP
S.l.: Saccharina latissima
S.l.-SP: sulfated polysaccharides extracted from Saccharina latissima
SGF: sulfated galactofucan
SP: sulfated polysaccharides
