# Supplementary material for: Sulfated Galactofucan from the Brown Alga Saccharina latissima—Variability of Yield, Structural Composition and Bioactivity
Source: Mar Drugs. 2014 Dec 26;13(1):76–101. doi: 10.3390/md13010076 (PMC4306926; doi:10.3390/md13010076)
Supplement: Supplementary File 1 [file marinedrugs-13-00076-s001.pdf]

Supplementary Information

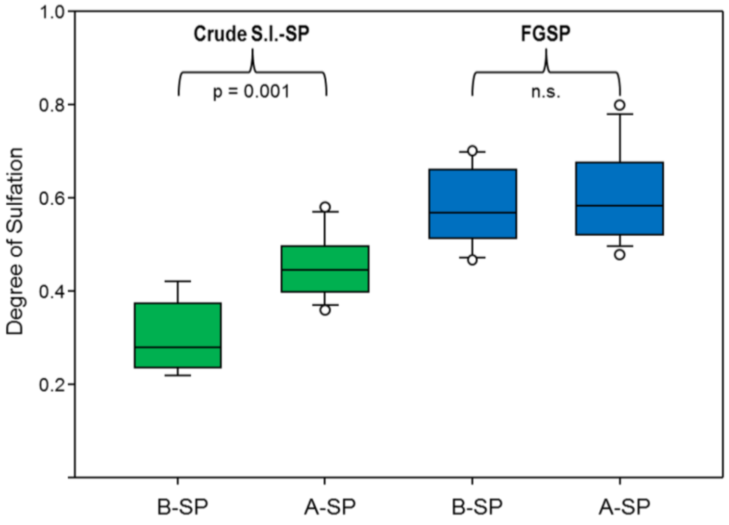

Figure S1. DS of Crude S.l.-SP and their FGSP Proportion in Relation to the Habitats Baltic Sea and Atlantic Ocean. The boxplots of B-SP represent the DS of the B05-SP and B06-SP batches; the boxplots of A-SP represent the DS of the A05-SP and A09-SP batches.

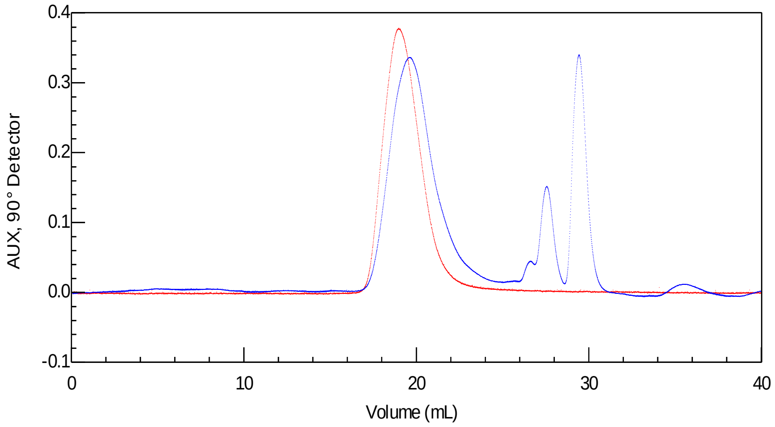

Figure S2. Size Exclusion Chromatogram of Fraction F3 of A09-SP (*i.e.*, Sulfated Galactofucan) (red line: M_r (MALLS); blue line: M_{HV} (RI)). A09-SP-F3 revealed a single peak with a mean M_r of 416,000 ± 28,000 (MALLS) and 449,000 ± 15, 000 (RI), respectively. The further peaks at about 28 mL are due to solvent effects.

Table S1. Methylation Analysis Data of Native and Desulfated A09-SP-F3 [a,b,c].

Glycosyl Residue	Position of *O*-Methyl Group	Deduced Position of Substitution	A09-SP-F3 (native)	A09-SP-F3 (Desulfated)
**Fucosyl**	2,3,4	terminal	-	42.6
	2,3	4	-	1.2
	2,4	3	-	33.4
	3,4	2	23.8	2.2
	2	3,4	35.3	-
	4	2,3	15.5	3.8
		2,3,4	8.5	-
**Total Fucose**			83.1	83.1
**Galactosyl**	2,3,4,6	terminal	-	1.5
	2,4,6	3	3.3	3.7
	2,3,6	4	-	3.7
	2,3,4	6	-	5.3
	2,6	3,4	2.5	-
	2,4	3,6	8.5	-
	2,3	4,6	-	2.3
	2	3,4,6	2.3	-
**Total Galactose**			16.6	16.6
**Xylosyl**	2,3,4	terminal	<1	<1
	2,3/3,4	4/2	<1	<1

[a] The numbers indicate % (mol/mol) of partially methylated alditol acetates (PMAA); [b] Due to degradation and especially loss of fucose during desulfation, the fucose data of desulfated A09-SP-F3 have been upscaled to 83.1% and those for galactose and xylose accordingly downscaled for simpler interpretation. However, the methylation data on desulfated A09-SP-F3 have to be treated with caution; [c] All monosaccharides showed to be present in pyranose form.

© 2014 by the authors; licensee MDPI, Basel, Switzerland. This article is an open access article distributed under the terms and conditions of the Creative Commons Attribution license (http://creativecommons.org/licenses/by/4.0/).
